# Risk of SARS-CoV-2 infection and subsequent hospital admission and death at different time intervals since first dose of COVID-19 vaccine administration, Italy, 27 December 2020 to mid-April 2021

**DOI:** 10.2807/1560-7917.ES.2021.26.25.2100507

**Published:** 2021-06-24

**Authors:** Alberto Mateo-Urdiales, Stefania Spila Alegiani, Massimo Fabiani, Patrizio Pezzotti, Antonietta Filia, Marco Massari, Flavia Riccardo, Marco Tallon, Valeria Proietti, Martina Del Manso, Maria Puopolo, Matteo Spuri, Cristina Morciano, Fortunato (Paolo) D’Ancona, Roberto Da Cas, Serena Battilomo, Antonino Bella, Francesca Menniti-Ippolito, on behalf of the Italian Integrated Surveillance of COVID-19 study group, on behalf of the Italian COVID-19 vaccines registry

**Affiliations:** 1 Department of Infectious Diseases, Istituto Superiore di Sanità, Rome, Italy; 2 European Programme for Intervention Epidemiology Training (EPIET), European Centre for Disease Prevention and Control (ECDC), Stockholm, Sweden; 3 National Centre for Drug Research and Evaluation, Istituto Superiore di Sanità, Rome, Italy; 4 Department of Informatics, Istituto Superiore di Sanità, Rome, Italy; 5 DG-SISS, Ministero della Salute, Rome, Italy; 6 Department of Neuroscience, Istituto Superiore di Sanità, Rome, Italy; 7 Research Coordination and Support Service, Istituto Superiore di Sanità, Rome, Italy; 8 The members have been listed at the end of this article; 9 The members have been listed at the end of this article

**Keywords:** COVID-19, vaccination, pandemic, Italy

## Abstract

To assess the real-world impact of vaccines on COVID-19 related outcomes, we analysed data from over 7 million recipients of at least one COVID-19 vaccine dose in Italy. Taking 0–14 days post-first dose as reference, the SARS-CoV-2 infection risk subsequently decreased, reaching a reduction by 78% (incidence rate ratios (IRR): 0.22; 95% CI: 0.21–0.24) 43–49 days post-first dose. Similarly, hospitalisation and death risks decreased, with 89% (IRR: 0.11; 95% CI: 0.09–0.15) and 93% (IRR: 0.07; 95% CI: 0.04–0.11) reductions 36–42 days post-first dose. Our results support ongoing vaccination campaigns.

On 27 December 2020, Italy launched its vaccination campaign against coronavirus disease (COVID-19) subsequent to approval of the mRNA Comirnaty vaccine (BNT162b2 mRNA, BioNTech-Pfizer, Mainz, Germany/New York, United States (US)). The mRNA Moderna COVID-19 Vaccine (mRNA-1273, Moderna, Cambridge, US) was approved on 14 January 2021, followed by the viral vector vaccine Vaxzevria (ChAdOx1 nCoV-19, Oxford-AstraZeneca, Cambridge, United Kingdom) on 2 February 2021 and the viral vector COVID-19 Vaccine Janssen (Ad26.COV2-S (recombinant), Janssen-Cilag International NV, Beerse, Belgium) on 12 March 2021 [1]. In the early stages, the campaign prioritised healthcare workers, residents in long-term care facilities (LCFT) and persons aged over 80 years [2]. The purpose of this study is to assess risk of severe acute syndrome respiratory coronavirus 2 (SARS-CoV-2) infections, as well as related hospitalisations and deaths following COVID-19 vaccination in Italy.

## Retrieving data and measuring the impact of vaccination in Italy

We conducted a cohort study among vaccinated individuals, aged 16 years and over, by linking COVID-19 vaccinated persons listed in the national vaccination registry of the Ministry of Health (14,365,241 on 3 May 2021), with data on positive cases of SARS-CoV-2 infection and related hospitalisations and deaths from the COVID-19 integrated surveillance system of the National Institute of Health, which we extracted on 2 May 2021 [3]. We excluded 567,162 (3.9%) cases who were infected before being vaccinated and 76,573 (0.5%) with missing information (Supplementary Material 1). Of the remaining 13,721,506 persons, 8,389,595 (61%) had received at least one dose of Comirnaty, 4,234,983 (31%) of Vaxzevria, 1,021,134 (7%) of Moderna and 75,794 (0.6%) of Janssen. Individuals vaccinated with COVID-19 Vaccine Janssen were not included in the further analyses conducted in this study. Almost all study participants followed standard protocols of dose administration for each specific vaccine: 95% (5,049,606/5,298,025) received the second dose within 21–25 days from the first dose of Comirnaty, 95% (412,850/433,785) within 28–31 days from the first dose of Moderna, and 95% (3,908/4,120) within 65–78 days from the first dose of Vaxzevria. In our analysis, 5,735,930 (42%) participants received a second dose by 3 May. The follow-up time of each participant was measured from the date of the first dose administration up to the end of follow-up, independently of the possible administration of the second dose. We did not exclude persons who received the second dose of vaccine and we did not censor anyone at the second dose administration.

**Figure 1:**
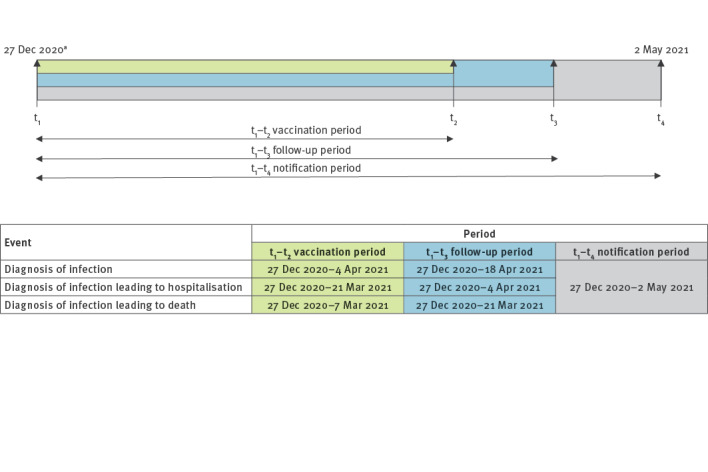
Timeline since the start of the vaccination campaign^a^ with the periods of selection and event ascertainment in the study population, Italy, 27 December 2020–2 May 2021

SARS-CoV-2: severe acute respiratory syndrome coronavirus 2; t: time.

^a^ The vaccination campaign started on 27 December 2020.

We analysed incidence rates of SARS-CoV-2 infection in 7,370,008 persons who had received a first dose of vaccine by 4 April 2021. Data were extracted on 2 May 2021, to allow for at least 2 weeks of follow-up after vaccination and another 2 weeks of possible delays in notification of the infection to the surveillance system. To measure hospitalisation rates, we included persons who had received a first dose of vaccine by 21 March 2021 (n = 5,133,899), to allow for a longer observation period, considering that a possible worsening of symptoms and hospitalisation may occur sometime after diagnosis, and for time to report the occurrence of hospital admission to the surveillance system. For the same reasons, we measured death rates by including all persons vaccinated by 7 March 2021 (n = 3,622,029) (Figure 1).

To measure the impact of vaccination, we calculated rates (as ratios of numbers of events to person-days of follow-up) of infection, hospitalisation and death in two periods: days 0–14 post-first dose and from 15 days after the first dose until the end of follow-up. In line with current scientific evidence, we considered rates in the first 14 days after the first dose as reference, assuming that during this period the risk of acquiring the infection is comparable to the unvaccinated population [4-6]. We then calculated incidence, hospitalisation and death rates by 7-day time-intervals until the end of follow-up. Hospitalisations and deaths were assigned to the dates when diagnosis of infection with SARS-CoV-2 occurred, and not to the period in which these events took place, given that we aimed to measure rates of infections leading to hospitalisation/death, not the time when these events per se took place.

Finally, we carried out a multivariable analysis on individual data split by period (i.e. 0–14 days and subsequent weekly time-intervals from the first dose). We used Poisson regression models, with robust variance estimation, to calculate incidence rate ratios (IRRs) with 95% confidence intervals (CI) measuring changes in the risk of infection, hospitalisation and death over different time periods after vaccination, compared with the initial 14-day period. To consider only events associated with COVID-19, we adopted a conservative approach including only hospitalisations and deaths occurring within 30 days from diagnosis of SARS-CoV-2 infection, which amount, respectively, to 99% (366,921/372,268) and 89% (107,671/121,075) of all those registered in the surveillance database who were hospitalised or died from the start of the epidemic in Italy (20 February 2020) until 2 May 2021. We included sex, age group, region of vaccination, vaccine brand, and vaccination priority group as covariates. Estimates were also adjusted for calendar week of administration of first vaccine dose and for regional weekly incidence in the general population to consider time-related and geographical variations in the probability of infection.

## Ethical statement

The dissemination of COVID-19 surveillance data was authorised by the Italian Presidency of the Council of Ministers on 27 February 2020 (Ordinance no. 640).

## SARS-CoV-2 infection, related hospitalisation and death rates in the first 14 days after first COVID-19 vaccine dose and afterwards

Compared with the reference period, we observed a reduction in the overall incidence of infection (2.9 vs 1.3 per 10,000 person-days), hospitalisation (0.44 vs 0.12) and death (0.18 vs 0.04), both overall and stratifying by age group, sex, geographical location and calendar period (Table).

**TABLE:** Rates of SARS-CoV-2 infection, related hospitalisation and death 0–14 days after administration of first COVID-19 vaccine dose and thereafter, Italy, 27 December 2020–18 April 2021

Characteristics of persons involved in the study		Total vaccinated	Period from administration of first dose						p value^a^
			0–14 days			> 14 days			
			Person-days	Events	Rate (per 10,000 person-days)	Person-days	Events	Rate (per 10,000 person-days)	
**SARS-CoV-2 infection**									
**Total**		7,370,008	102,991,407	29,839	2.90	240,605,533	32,020	1.33	< 0.001
**Age group in years**	< 40	1,098,052	15,334,867	5,605	3.66	48,764,109	6,143	1.26	< 0.001
	40–59	2,042,446	28,529,032	10,102	3.54	85,752,132	11,962	1.39	< 0.001
	60–79	1,714,155	23,966,168	5,141	2.15	41,622,567	4,479	1.08	< 0.001
	≥ 80	2,515,355	35,161,340	8,991	2.56	64,466,725	9,436	1.46	< 0.001
**Sex**	Males	3,055,506	42,700,832	12,039	2.82	95,747,599	11,835	1.24	< 0.001
	Females	4,314,502	60,290,577	17,800	2.95	144,857,934	20,185	1.39	< 0.001
**Date of immunisation with a first dose of COVID-19 vaccine**	27 Dec–15 Jan	1,011,356	14,100,876	8,555	6.07	85,029,552	11,088	1.30	< 0.001
	16 Jan–30 Jan	229,767	3,206,796	1,484	4.63	16,973,189	2,177	1.28	< 0.001
	31 Jan–14 Feb	340,052	4,753,120	1,264	2.66	17,962,690	2,086	1.16	< 0.001
	15 Feb–1 Mar	1,275,340	17,827,593	4,625	2.59	50,920,224	6,654	1.31	< 0.001
	2 Mar–16 Mar	1,831,139	25,592,249	7,098	2.77	48,233,854	7,228	1.50	< 0.001
	17 Mar–28 Mar	1,512,076	21,142,756	4,223	2.00	17,657,048	2,288	1.30	< 0.001
	29 Mar–4 Apr	1,170,278	16,368,017	2,590	1.58	3,828,976	499	1.30	< 0.001
**COVID-19 hospitalisation**									
**Total**		5,133,899	71,852,063	3,175	0.44	155,833,205	1,797	0.12	< 0.001
**Age group in years**	< 40	890,565	12,466,598	93	0.07	35,107,107	33	0.01	< 0.001
	40–59	1,643,782	23,009,741	353	0.15	60,464,621	144	0.02	< 0.001
	60–79	812,377	11,369,440	525	0.46	25,025,547	239	0.10	< 0.001
	≥ 80	1,787,175	25,006,284	2,204	0.88	35,235,931	1,381	0.39	< 0.001
**Sex**	Males	2,077,697	29,076,046	1,688	0.58	60,964,774	835	0.14	< 0.001
	Females	3,056,202	42,776,017	1,487	0.35	94,868,431	962	0.10	< 0.001
**Date of immunisation with a first dose of COVID-19 vaccine**	27 Dec–15 Jan	1,069,755	14,153,395	585	0.41	71,531,426	423	0.06	< 0.001
	16 Jan–30 Jan	171,252	3,215,357	152	0.47	13,874,842	123	0.09	< 0.001
	31 Jan–14 Feb	314,180	4,759,689	99	0.21	13,309,313	100	0.08	< 0.001
	15 Feb–1 Mar	1,126,326	17,849,208	759	0.43	33,370,730	552	0.17	< 0.001
	2 Mar–16 Mar	2,005,929	25,626,251	1,245	0.49	22,882,081	558	0.24	< 0.001
	17 Mar–28 Mar	446,457	6,248,165	335	0.54	864,813	41	0.47	0.464
**COVID-19 death**									
**Total**		3,622,029	50,701,430	899	0.18	94,439,262	418	0.04	< 0.001
**Age group in years**	< 40	714,934	10,008,516	0	0.00	23,815,086	0	0.00	NC
	40–59	1,270,264	17,783,149	7	0.00	39,777,616	8	0.00	0.207
	60–79	533,419	7,466,957	111	0.15	15,939,429	38	0.02	< 0.001
	≥ 80	1,103,412	15,442,809	781	0.51	14,907,130	372	0.25	< 0.001
**Sex**	Males	1,437,537	20,122,280	443	0.22	36,295,566	167	0.05	< 0.001
	Females	2,184,492	30,579,151	456	0.15	58,143,696	251	0.04	< 0.001
**Date of immunisation with a first dose of COVID-19 vaccine**	27 Dec–15 Jan	1,069,850	14,156,286	360	0.25	57,579,446	192	0.03	< 0.001
	16 Jan–30 Jan	171,272	3,216,218	78	0.24	10,696,048	53	0.05	< 0.001
	31 Jan–14 Feb	314,195	4,760,332	42	0.09	8,571,541	32	0.04	< 0.001
	15 Feb–1 Mar	1,126,356	17,852,787	260	0.15	15,555,615	117	0.08	< 0.001
	2 Mar–16 Mar	940,356	10,715,807	159	0.15	2,036,612	24	0.12	0.293

COVID-19: coronavirus disease; SARS-CoV-2: severe acute respiratory syndrome coronavirus 2; NC: not calculable.

^a^ Mid p values adjusted test of incidence-rate difference.

Reduction in the adjusted risk of infection, hospitalisation and death in vaccinated persons by 7-days with respect to the first 14 days post-first COVID-19 vaccine dose.

**Figure 2:**
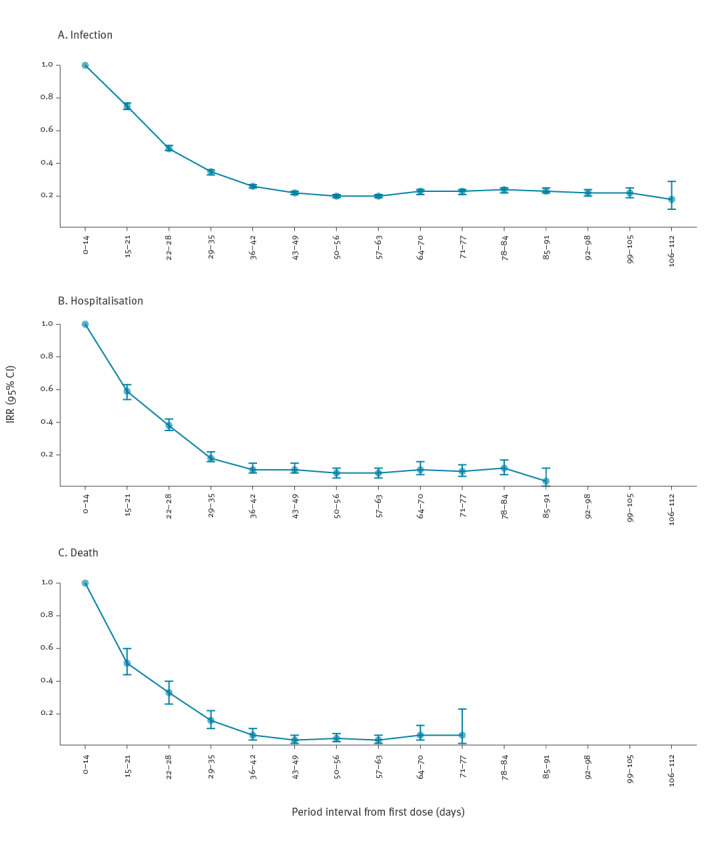
Adjusted incidence rate ratios with 95% confidence intervals of SARS-CoV-2 (A) infection, (B) hospitalisation and (C) death, by 7-day period with respect to the first 14 days post first-dose of COVID-19 vaccine**, **Italy, 27 December 2020–18 April 2021

CI: confidence interval; COVID-19: coronavirus disease; IRR: incidence rate ratio; SARS-CoV-2: severe acute respiratory syndrome coronavirus 2.

The adjusted risk of SARS-CoV-2 infection decreased gradually until the period 42–49 days post-first dose, with a reduction of up to 78% (IRR: 0.22; 95% CI: 0.21–0.24). Subsequently, the risk level stabilised. Similarly, the risk of hospitalisation and death among reported COVID-19 cases decreased gradually until the period 35–42 days post-first dose, when the estimated risk reduction was 89% (IRR: 0.11; 95% CI: 0.09–0.15) for hospitalisation and 93% (IRR: 0.07; 95% CI: 0.04–0.11) for death. After this period, the risks remained stable (Figure 2).

**Figure 3:**
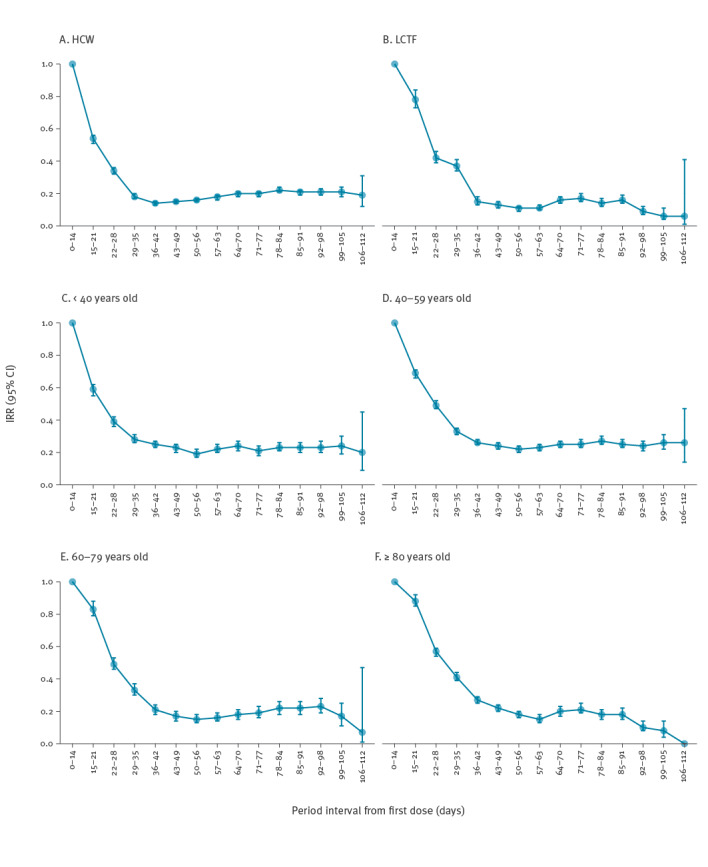
Adjusted incidence rate ratios with 95% confidence intervals of SARS-CoV-2 infection in (A) healthcare workers, (B) residents of long-term care facilities and (C–F) age groups, by 7-day period with respect to the first 14 days post first-dose of COVID-19 vaccine, Italy, 27 December 2020–18 April 2021

CI: confidence interval; COVID-19: coronavirus disease; HCW: healthcare worker; IRR: incidence risk ratios (adjusted); LTCF: long-term care facility residents, SARS-CoV-2: severe acute respiratory syndrome coronavirus 2.

The analyses by age and priority group confirm a gradual decrease in the risk of infection from 14 to 42 days after first dose with an approximate risk reduction of 80% compared with the first 14 days post-first dose. Towards the end of the follow-up period (99–105 days post-first dose), the risk reduction was significantly more pronounced in residents in LCTF (IRR: 0.06; 95% CI: 0.04–0.11) than in HCWs (IRR: 0.21; 95% CI: 0.18–0.24). Equally, during the same period, the risk reduction in those aged over 80 years of age (IRR: 0.08; 95% CI: 0.04–0.14) was significantly higher than in those aged 40-59 years (IRR: 0.26; 95% CI: 0.22–0.31) and aged 40 years or less (IRR: 0.24; 95% CI: 0.19–0.30) (Figure 3).

## Discussion

Our results suggest a significantly reduced risk of SARS-CoV-2 infection, and COVID-19 related hospitalisation and death in vaccinated individuals from 2 weeks post-vaccination with a first dose compared with the period comprising the first 2 weeks following vaccination. Risk reduction gradually increased from week 2 post-first dose until weeks 5–6, after which it remained stable.

We analysed over 7 million vaccinated people, the largest cohort used to date, to assess the effect of COVID-19 vaccines. This allowed us to adjust the estimates for several possible confounding factors and to stratify by age and priority groups.

Results agree with previous reports in finding COVID-19 vaccines to be effective in preventing SARS-CoV-2 infection. Four studies focused on the Comirnaty vaccine reported risk reduction estimates of 85–95% 1 week after second dose [7-10] and a study including both Comirnaty and Moderna found a 94% risk reduction in persons aged 65 years and older [11]. With regards to hospitalisations, risk reductions of up to 87% with Comirnaty and 84% with Comirnaty and Vaxzevria have been reported, which are similar to our 89% estimate [7,12]. Finally, evidence from Israel suggest that Comirnaty provides, from 1 week after the second dose, a 96.7% reduction of the risk of dying, similar to our estimate of 93% [9].

Unlike previous studies [9], we found higher risk reductions in older age groups than in the younger population, and in persons living in LCTF compared with HCW. These findings could have several explanations. As residents and staff of LCTF were among the first to be vaccinated, it is possible that residents in these facilities may have benefited from some degree of community protection, especially in a context of strict control of outside visitors. It is also possible, however, that estimates in this population, and in the older age groups, are biased towards overestimation of the risk reduction due to lack of survival ascertainment. As we only record deaths in SARS-CoV-2 positive individuals, persons dying from other causes – and therefore no longer at risk of infection – are still considered alive and therefore at risk.

Prior published studies used a control group of unvaccinated persons to estimate risk reduction, whereas we used the first 14 days post-first dose as the reference period. This approach has its strengths and limitations. Although studies have found that COVID-19 vaccines only start to provide significant protection after 2 weeks post-first dose [4-6], the immune response triggered by vaccination is gradual, and could already confer some degree of protection in the last days of this period. In this sense, for example, a study found a lower risk of infection in days 10–13 post-first COVID-19 vaccine dose compared with the previous period, though not significantly lower than in the unvaccinated population [8]. It is possible that our estimates of risk reduction are partially underestimating the protective effect of vaccines. As this study did not collect data on the unvaccinated population, it was not possible to conduct a sensitivity analysis with unvaccinated people as a control. On the other hand, being vaccinated in the first stages of the vaccination campaign could be influenced by strategical (e.g. high-risk groups), behavioural (e.g. vaccine hesitancy) and/or psychological (e.g. self-perceived risk) factors which could differ systematically between the vaccinated and the unvaccinated populations. These factors, if not accounted for, could introduce biases that might be avoided by using the same population as the reference group. To test the robustness of our results, we carried out a sensitivity analysis using the first 7 days post-first dose as the reference period. We found that during the period 7–14 days post-first dose, the risk of hospitalisation and death was similar to the reference period, but that the risk of infection was higher (Supplementary Material 2). One explanation for this finding is that vaccinated individuals may be less likely to respect social-distancing rules in the immediate days after vaccination [13]. As immunity is not yet complete during this period, this leads to a higher-than-expected infection rate. However, estimates of the risk reduction in the weekly time intervals from 14 days post-vaccination onwards were very close to those obtained using the whole period 0–14 days post vaccination as reference.

Furthermore, other factors could have affected the precision of our estimates. For example, it is possible that vaccinated individuals may be less likely to seek testing for SARS-CoV-2 infection if asymptomatic or only mildly symptomatic. As we only have information on cases notified through the surveillance system, this could also lead to an overestimation of our risk reduction estimates. Finally, we did not compare different brands or vaccines types (mRNA, viral vector), since different vaccines were introduced in different time periods, have different time schedules and were administered to population groups with differing risk profiles. A longer follow-up time is required for meaningful comparisons.

## Conclusion

Compared with the first 2 weeks after the first dose, the risk of infection, hospitalisation and death from SARS-CoV-2 decreases gradually and substantially in vaccinated individuals. These results suggest that the vaccination campaign has the potential to significantly reduce the burden of COVID-19 in terms of morbidity and mortality if high levels of vaccine uptake are achieved.

## Italian Integrated Surveillance of COVID-19 study group

ISS coordination group: Antonino Bella, Martina Del Manso, Xanthi Andrianou, Alberto Mateo Urdiales, Massimo Fabiani, Matteo Spuri, Stefania Bellino, Stefano Boros, Maria Cristina Rota, Ornella Punzo, Maria Fenicia Vescio, Daniele Petrone, Marco Tallon, Corrado Di Benedetto, Alessandra Ciervo, Paola Stefanelli, Flavia Riccardo, Patrizio Pezzotti.

Regional representatives: Antonia Petrucci (Abruzzo); Michele La Bianca (Basilicata); Anna Domenica Mignuoli (Calabria); Pietro Buono (Campania); Erika Massimiliani (Emilia-Romagna); Fabio Barbone (Friuli Venezia Giulia); Francesco Vairo (Lazio); Camilla Sticchi (Liguria); Danilo Cereda (Lombardia); Lucia Di Furia (Marche); Francesco Sforza (Molise); Annamaria Bassot (P.A. Bolzano); Pier Paolo Benetollo (P.A. Trento); Chiara Pasqualini (Piemonte); Lucia Bisceglia (Puglia); Maria Antonietta Palmas (Sardegna); Salvatore Scondotto (Sicilia); Emanuela Balocchini (Toscana); Anna Tosti (Umbria); Mauro Ruffier (Valle D'Aosta); Filippo Da Re (Veneto).

## Italian COVID-19 vaccines registry

Italian Ministry of Health: Serena Battilomo, Valeria Proietti.

Regional representatives: Camillo Odio (Abruzzo); Michele Recine (Basilicata); Innocenza Ruberto (Calabria); Salvatore Ascione e Massimo Bisogno (Campania); Gandolfo Miserendino, Massimiliano Navacchia (Emilia-Romagna); Beatrice Del Frate, Emanuela Cau (Friuli Venezia Giulia); Diego Baiocchi, Danilo Fusco (Lazio); Domenico Gallo (Liguria); Maria Rosa Marchetti (Lombardia); Liana Spazzafumo (Marche); Raffaele Malatesta (Molise); Antonio Fanolla (P.A. Bolzano); Diego Conforti, Carlo Trentini (P.A. Trento); Antonino Ruggeri (Piemonte); Concetta Ladalardo, Nehludoff Albano (Puglia); Marco Corona, Paolo Lombardi (Sardegna); Massimo Iacono (Sicilia); Paolo Bruno Angori, Andrea Belardinelli (Toscana); Milena Solfiti (Umbria); Stefano Fioraso (Valle D'Aosta); Chiara Poma, Nadia Raccanello (Veneto).

## Supplementary Data

624000 Supplementary_Material
