# Supplementary material for: Risk of SARS-CoV-2 infection and subsequent hospital admission and death at different time intervals since first dose of COVID-19 vaccine administration, Italy, 27 December 2020 to mid-April 2021
Source: Euro Surveill. 2021 Jun 24;26(25):2100507. doi: 10.2807/1560-7917.ES.2021.26.25.2100507 (PMC8229378; doi:10.2807/1560-7917.ES.2021.26.25.2100507)

This supplementary material is hosted by *Eurosurveillance* as supporting information alongside the article "Risk of SARS-CoV-2 infection and subsequent hospital admission and death at different time intervals since first dose of COVID-19 vaccine administration, Italy, 27 December 2020 to mid-April 2021" on behalf of the authors who remain responsible for the accuracy and appropriateness of the content. The same standards for ethics, copyright, attributions and permissions as for the article apply. Supplements are not edited by Eurosurveillance and the journal is not responsible for the maintenance of any links or email addresses provided therein.

Supplementary Material 1: Flowchart with the selection of the vaccinated population from the national vaccination registry and cases from the COVID-19 integrated surveillance system.

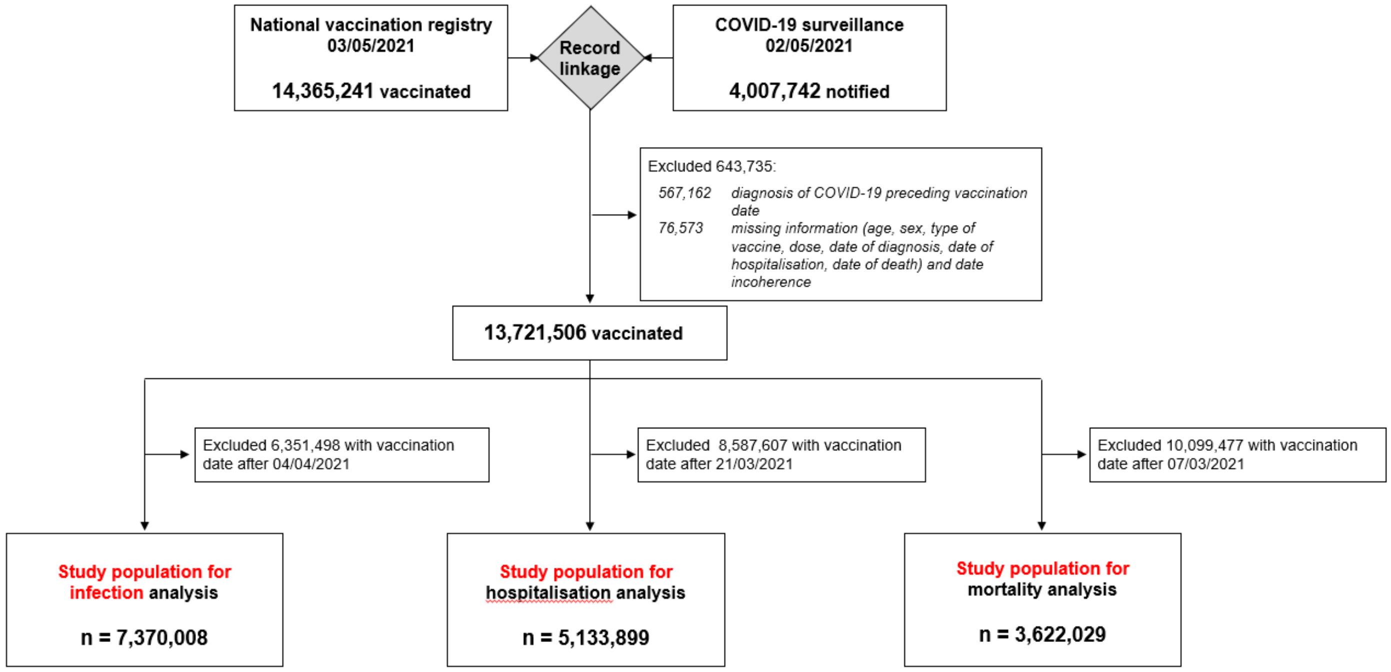

Supplementary Material 2. Sensitivity analysis. Adjusted incidence rate ratios with 95% confidence intervals of SARS-CoV-2 (A) infection, (B) hospitalisation and (C) death, by 7-day period with respect to the first 7 days post first-dose of COVID-19 vaccine, Italy, 27 December 2020–18 April 2021

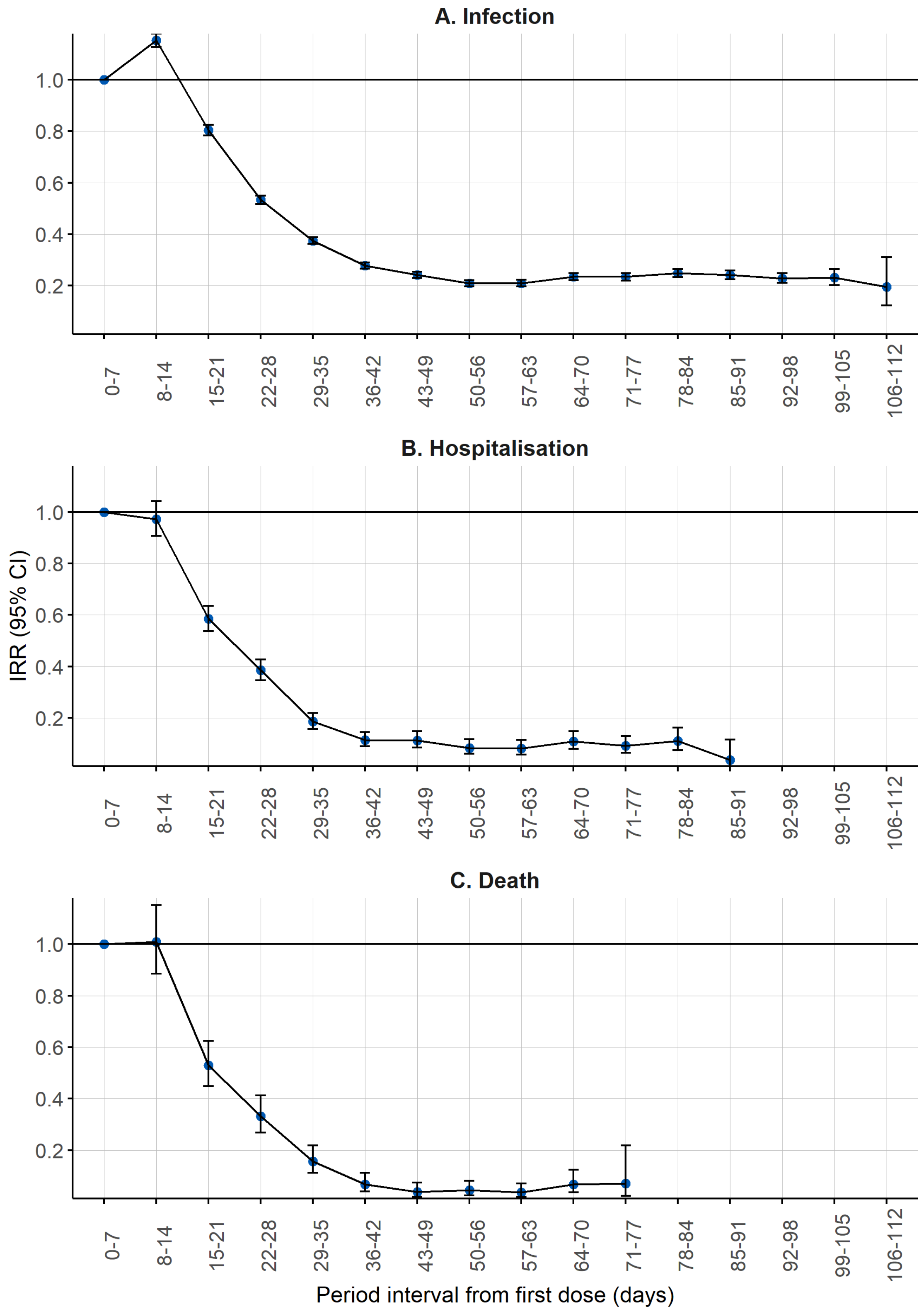

Supplement: Supplementary_Material [file 2100507_Supplementary_Material.pdf]
